# Molecular and Serological Surveillance of Mosquito-Borne Viruses in Racehorses or Mosquitoes From Horse Farms in Shanghai, China, 2022

**DOI:** 10.1155/tbed/6131435

**Published:** 2025-10-26

**Authors:** Yan Zhang, Jiayang Zheng, Hailong Zhang, Yafang Lin, Yan Wang, Zhiyong Ma, Jianchao Wei, Bin Zhou, Dengke Zhong

**Affiliations:** ^1^College of Veterinary Medicine, Nanjing Agricultural University, Nanjing 210095, China; ^2^Shanghai Veterinary Research Institute, Chinese Academy of Agricultural Sciences, Shanghai 200241, China; ^3^Technical Center for Animal, Plant and Food Inspection and Quarantine of Shanghai Customs, Shanghai 200135, China; ^4^Department of Animal Science and Technology, Shanghai Vocational College of Agriculture and Forestry, Shanghai 201699, China

**Keywords:** molecular, mosquito-borne viruses, mosquitoes, racehorses, seroprevalence

## Abstract

Getah virus (GETV), Japanese encephalitis virus (JEV), West Nile virus (WNV), and African horse sickness virus (AHSV) are mosquito-borne viruses threatening the health of racehorses. However, the systematic surveillance of these viruses among Shanghai racehorses remains lacking. Therefore, molecular and serological surveillance was conducted for these viruses in racehorses and mosquitoes at horse farms in Shanghai, China, during 2022 to assess their prevalence. Among 11,140 mosquitoes collected from seven farms across four districts, *Culex tritaeniorhynchus* and *Anopheles sinensis* were identified as the dominant species. RT-qPCR detected GETV in four mosquito pools (FX1-6, PD1-32, PD1-45, and PD1-57) and JEV in three pools (SJ1-4, PD1-22, and JS1-9), while WNV and AHSV remained undetected. Two GETV strains (SH202201 and SH202202) were isolated and phylogenetically classified as genotype III (GIII). Serological surveys of 182 horse serum samples revealed an overall GETV antibody positivity rate of 28.6%. The positivity rate demonstrated significant age-dependency (41.7% in horses >15 years) and seasonal variation (45.1% in autumn vs. 12.1% in spring). JEV seroprevalence rates were 12.6%, exhibiting significant seasonal differences. No antibodies positive for WNV and AHSV were detected. These results indicate that the threat of WNV and AHSV to racehorses in Shanghai is currently very small, while GETV represents the primary arboviral risk. Implementing targeted surveillance for GETV during high-risk seasons (autumn) and in key regions (Fengxian), while enhancing surveillance for JEV, WNV, and AHSV, is crucial for safeguarding equine health and promoting the sustainable development of the equestrian industry.

## 1. Introduction

Mosquito-borne viruses pose substantial threats to global livestock industries and public health. These viruses frequently cause acute or chronic diseases in equine populations, leading to encephalitis, diminished athletic performance, reproductive disorders, and even mortality. Such infections impose substantial economic losses on the racing industry and destabilize the equestrian sector. Common mosquito-borne viruses threatening racehorse health include Getah virus (GETV), Japanese encephalitis virus (JEV), West Nile virus (WNV), and African horse sickness virus (AHSV). Currently, no specific antiviral therapies exist for these pathogens; the primary protective measures remain the vaccination of susceptible hosts, vector control, and enhanced surveillance. As China's pivotal equestrian hub hosting high-level international competitions with extensive racehorse populations, Shanghai requires the rigorous monitoring of mosquito-borne viruses within its horse farms to safeguard equine health and ensure sustainable industry development.

Notably, GETV now severely threatens the sustainable development of the equine and swine industries both in China and globally, inflicting substantial economic losses on livestock production. GETV is a member of the *Alphavirus* genus within the *Togaviridae* family [1]. It is a mosquito-borne virus that was first isolated from *Culex* mosquitoes in Malaysia in the 1950s [2]. It has been detected in various mosquito species, including *Aedes vexans*, *Armigeres obturbans*, *Armigeres subalbatus*, and *Anopheles sinensis* [3]. GETV is widely distributed across Asia and Oceania, including China, South Korea, Japan, Russia, Thailand, Malaysia, India, Australia, the Philippines, Cambodia, and Vietnam [4–7]. In China, since the first isolation of GETV from mosquitoes in Hainan Province in 1964, it has spread to 17 provinces and cities [8]. GETV is an emerging animal pathogen; it has been detected in feverish cattle and blue foxes with neurological symptoms[3, 9]. Furthermore, neutralizing antibodies against GETV have been identified in human serum [10]. Racehorses and pigs serve as the primary amplifying and reservoir hosts for GETV, playing a crucial role in the transmission dynamics of the virus. In pigs, GETV can cause reproductive disorders in sows and severe diarrhea or even death in piglets [11], whereas in racehorses, GETV infection can lead to fever, anorexia, and edema in the hind limbs [12]. Several outbreaks of GETV infection have occurred in racehorses in Japan and India and have resulted in large economic losses [13].

In addition to GETV, other mosquito-borne viruses, including WNV, JEV, and AHSV, also represent substantial threats to equine health[14, 15]. AHSV, the etiological agent of African horse sickness (AHS), is primarily transmitted by *Culicoides* midges but can also be spread by other hematophagous vectors such as ticks and mosquitoes [16]. AHS is a notifiable disease listed by the World Organization for Animal Health (WOAH) [17]. Its clinical manifestations range from mild fever to severe acute forms, with mortality rates being >95% in fully susceptible domestic horses [18]. From the perspective of viral transmission ecology, racehorses generally serve as terminal hosts for WNV and JEV because viral replication in their bodies fails to induce significant viremia, thus interrupting the transmission chain from vertebrate hosts to mosquito vectors [19]. Nevertheless, the low-level viremia triggered by WNV and JEV in infected racehorses can still lead to sporadic cases in equine populations, with clinical manifestations including fever, meningitis, encephalitis, and flaccid paralysis [20]. It is noteworthy that racehorses often act as sentinel animals for active viral circulation in serological surveillance systems for WNV and JEV, as changes in their antibody positivity rates can reflect the dynamics of viral transmission in the region [21, 22].

As the economic, financial, and trade center of China, Shanghai has become a benchmark city for China's equestrian industry owing to its economic strength, international perspective, and high-end consumer market. With the increasing number of international equestrian events held in Shanghai, monitoring and controlling the prevalence of mosquito-borne viruses to which racehorses are susceptible—such as JEV, WNV, GETV, and AHSV—have become of paramount importance, as these viruses pose significant threats to the health of racehorses in the region. Therefore, this study conducted an epidemiological investigation into JEV, WNV, GETV, and AHSV infections in racehorses and the mosquito vectors at horse farms in Shanghai. The aim was to assess the prevalence of these viruses in Shanghai and implement timely intervention and control measures. This is of great significance for safeguarding equine health, ensuring the smooth holding of international equestrian events, promoting the sustainable development of the horse racing industry, and protecting regional economic interests.

## 2. Materials and Methods

### 2.1. Sample Collection and Detection

#### 2.1.1. Mosquito Samples

**Figure 1:**
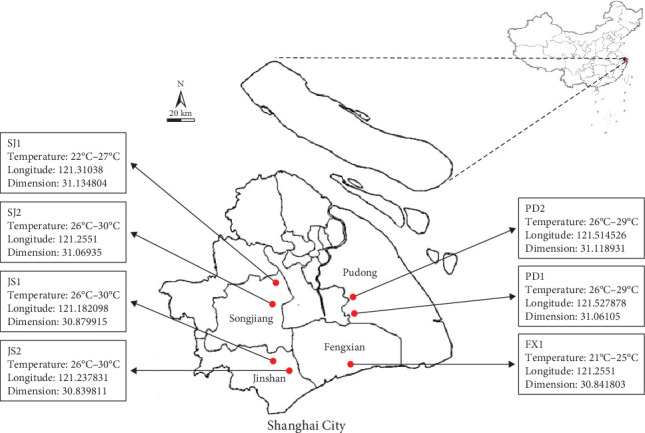
The geographical location of the mosquito collection points on horse farms in Shanghai, China.

In 2022, mosquitoes were collected at seven large horse farms in four districts in Shanghai (Pudong, Fengxian, Jinshan, and Songjiang) using mosquito suction devices and CDC-type traps. The specific sampling location information is presented in Figure 1. The collected mosquitoes were morphologically identified on ice. They were grouped and counted according to species, with 100 mosquitoes placed in 2 mL EP tubes and stored in a −80°C freezer. All the mosquito pool samples were resuspended in a 0.9% sodium chloride solution (Shanghai Xinyu Biotechnology Co., Ltd.). Total RNA was isolated from 200 μL homogenized samples using a magnetic bead-based nucleic acid extraction kit (Hangzhou Bioer Technology Co., Ltd.) according to the manufacturer's instructions. Subsequently, the purified RNA was reverse-transcribed into cDNA using the Evo M-MLV Reverse Transcription Kit (Accurate Biotechnology Co., Ltd.) following the manufacturer's recommended protocol. The cDNA products were analyzed by RT-qPCR using specific primers targeting the E2 gene of GETV, the NS1 gene of JEV, the NS5 gene of WNV, and the NS1 gene of AHSV [23–25] (Table 1).

The mosquito pools identified as positive by RT-qPCR were inoculated into BHK-21 cells. After 48 h, cytopathic effects were observed, and the virus was blind-passaged for three generations. Cell culture supernatants were collected, and viral RNA was extracted. RT-PCR detection of the E2 gene was performed using GETV-specific primers (forward primer: 5′-AGTGTGACGGAACACTTCAATGTTAC-3′; reverse primer: 5′-GGCATGCGCTCGTGGT-3′). The amplicons were purified and sequenced (Sangon Biotech [Shanghai] Co., Ltd.).

#### 2.1.2. Horse Serum Samples

Whole-blood samples were collected from racehorses at four racetracks in Shanghai in the spring (March) and autumn (September) of 2022. All serum samples were separated by centrifugation and stored at −20°C for later analysis. A total of 182 horse serum samples were collected and classified according to the horse's age, gender, collection region, and season (Table 2). Serological testing utilized ELISAs as follows: GETV was detected using a previously established in-house ELISA [26]; WNV antibodies were analyzed with the Horse WNV Antibody ELISA Kit (Qingdao Real Bio-Technology Co., Ltd.); JEV antibodies were examined using the Horse JEV Antibody ELISA Kit (Shanghai Cosun Biotechnology Co., Ltd.); and AHSV antibodies were assessed with the AHSV Antibody ELISA Kit (Shanghai Canyou Industrial Co., Ltd.). All procedures adhered strictly to the manufacturers' protocols.

### 2.2. Phylogenetic Analysis and Amino Acid Alignment

The MEGA software (Version 11.0.13, Mega Limited, New Zealand) was used for sequence analysis. The phylogenetic tree was constructed using MEGA 11 (New Zealand), based on multiple GETV sequences from GenBank, with 1000 bootstrap replicates and the Neighbor-Joining method. To identify mutations in the E2 gene, an amino acid difference analysis was performed by comparing the obtained sequences with the reference sequence.

## 3. Results

### 3.1. Identification of Mosquitoes on Horse Farms in Shanghai

To understand the prevalence of GETV on horse farms in Shanghai and the species composition of its vector mosquitoes, we collected mosquitoes from seven large horse farms across four districts of Shanghai in 2022. A total of 11,140 mosquitoes belonging to six species within three genera (10,738 *Culex tritaeniorhynchus* [96.39%], 116 *Culex quinquefasciatus* [1.04%], and 249 *Anopheles sinensis* [2.24%]) were collected during July 2022 (Table 3). Species belonging to the *Culex* genus were the most dominant, with 10,854 mosquitoes, accounting for 97.43% of the total mosquito collection. The 11,140 mosquitoes were divided into 123 sample pools according to species and collection location (Table 4) and preserved at −20°C.

### 3.2. Detection of Three Viruses in Mosquito Pool Samples at Shanghai Horse Farms

RT-qPCR analysis revealed GETV positivity in mosquito pools FX1-6, PD1-32, PD1-45, and PD1-57 (Ct < 35). JEV was additionally detected in pools SJ1-4, PD1-22, and JS1-9 (Ct < 35). Meanwhile, WNV and AHSV were undetected in all mosquito samples collected from equine farms during this study (Table 5).

### 3.3. Phylogeny Based on the GETV E2 Gene and Analysis of Critical Amino Acid Residues

**Figure 2:**
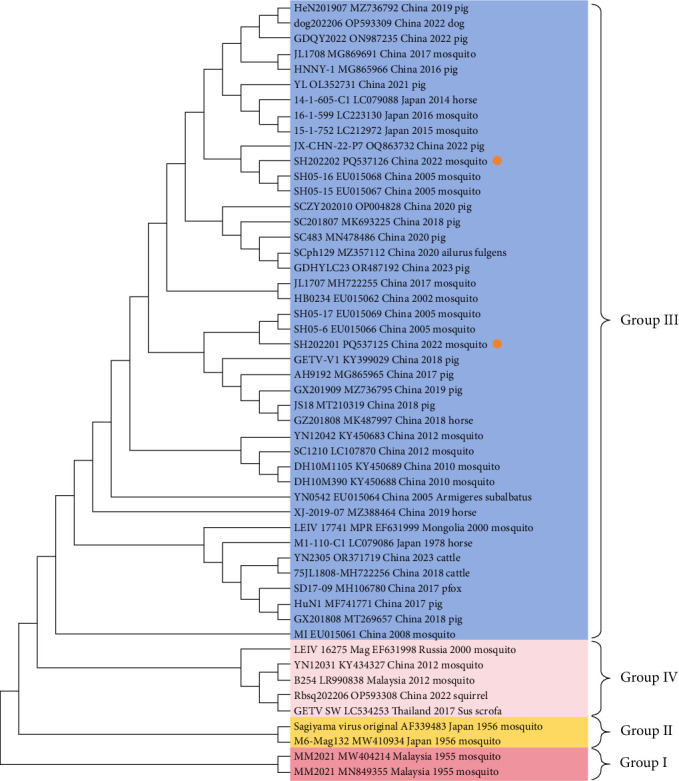
Phylogenetic analysis of GETV E2 gene nucleotide sequences isolated in Shanghai in 2022. The strain isolated in this study is identified by a solid circle (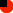).

Virus isolation was performed using six RT-qPCR-positive mosquito pool specimens. Only two GETV-positive samples, SH202201 and SH202202, caused the CPE phenomenon after cell inoculation. Full-length E2 gene sequences of SH202201 and SH202202 were obtained through Sanger sequencing. GETV has been classified into four major phylogenetic groups: Group I (GI), GII, genotype III (GIII), and genotype IV (GIV). Phylogenetic analysis based on the E2 gene sequence indicated that GETV SH202201 (PQ537125) and SH202202 (PQ537126) are located in the same evolutionary branch as the previously isolated Shanghai strains SH05-6 (EU015066) and SH05-16 (EU015068), showing the closest genetic relationship. All these strains belong to GIII GETV, with the conserved amino acid sequences and nucleotide sequence homology exceeding 99.8%. These strains are more distantly related to the GI Malaysian prototype strain MM2021 (MN849355) at the root of the evolutionary branch, with homology of only 93.6% and 94.2%, and 12 differing amino acid sites. Although SH202201 and SH202202 are distantly related to GⅡ, they lack mutations at some key amino acid sites. In fact, GETV strains prevalent in China mainly belong to GIII. GIV GETV strains (OP593308 and KY434372) were isolated in 2012 and 2022, with homology to SH202201 and SH202202 ranging from 94.9% to 96.4%, and multiple amino acid differences (Table 6 and Figure 2). The emergence of GIV GETV suggests that GETV strains circulating in China are gradually diversifying. Therefore, strengthening the monitoring and epidemiological investigation of GETV is becoming increasingly important for preventing and controlling its spread. In addition, the SH202201 and SH202202 strains isolated from mosquitoes had varying degrees of amino acid mutations in comparison to the E2 gene of GETV strains isolated from pigs and racehorses (Table 6). This suggests that GETV strains circulating in different mosquito species exhibit a certain degree of species specificity.

### 3.4. GETV, JEV, WNV, and AHSV Serological Epidemiological Survey

We also collected 182 horse serum samples from the horse farms to test for GETV, JEV, WNV, and AHSV infection. Samples were categorized by racehorse age, gender, collection season, and geographical region for statistical analysis.

Serological analysis by ELISA revealed GETV antibodies in 28.6% (52/182) of horse sera, while JEV seroprevalence was 12.6% (23/182). Notably, all equine serum samples tested negative for WNV and AHSV antibodies, which is consistent with the absence of WNV and AHSV RNA in the mosquito pool samples. This indicates that there was no active circulation of WNV or AHSV among Shanghai's equine population during the study period.

Age-stratified analysis revealed a distinct upward trend in the seroprevalence of both GETV and JEV, with rates of 31.6% and 10.5% in the 1–5 years age group, 23.1% and 13.5% in the 5–10 years group, 25.0% and 13.9% in the 10–15 years group, and 41.7% and 25% in the >15 years group, respectively. This age-dependent pattern indicates active circulation of GETV and JEV within Shanghai's equine population, with advanced age conferring an increased risk of infection (Table 2).

Gender-specific seroprevalence analysis revealed no statistically significant difference in GETV antibody positivity between male (27.5%) and female (29.2%) racehorses (*p*  > 0.05). Similarly, JEV seropositivity showed minimal variation (males: 13.0%; females: 12.4%). These findings indicate no gender-dependent patterns in susceptibility to GETV and JEV infections among Shanghai's equine population (Table 2).

Shanghai's mosquito breeding season (June–September) constitutes the peak period for arboviral transmission. Comparative seroprevalence analysis revealed that the antibody levels of both GETV and JEV in September were significantly higher than those in March. Specifically, their seropositivity rates were markedly elevated in autumn (45.1% and 18.7%) compared to spring (12.1% and 6.6%). These pronounced seasonal fluctuations indicate a strong temporal alignment between equine infections and peak mosquito activity, with autumn representing a high-risk period for arboviral transmission among Shanghai's equine population (Table 2).

Significant geographical variations were observed in seroprevalence. GETV positivity rate was highest in Fengxian District (42.5%), significantly exceeding that in Pudong (15.6%), Songjiang (22.9%), and Jinshan (30.6%) (*p* ≤ 0.05). No regional differences were noted for JEV positivity (Table 2).

## 4. Discussion

GETV has been widely identified in mosquitoes, pigs, foxes, and cattle across a broad geographic region. Notably, Japan and India have reported horse infections with GETV, causing a significant economic impact. GETV was first isolated from blood samples of febrile racehorses in Guangdong Province, China, in 2018. This highlights the need to monitor the prevalence and transmission dynamics of GETV in horse populations in China. As a mosquito-borne pathogen, in addition to enhancing epidemiological surveillance in horse populations, it is essential to systematically monitor the species composition of mosquito vectors and their GETV carriage status in surrounding ecosystems. In addition to GETV, this study simultaneously monitored JEV, WNV, and AHSV in mosquitoes and racehorses, as these arboviruses pose recognized threats to equine health in Shanghai's equestrian context.

Therefore, this study comprehensively analyzed the ecology and evolution of GETV in Shanghai by integrating mosquito vector species composition, viral genetic characteristics, and patterns of equine serological infections. Our mosquito collections from racecourses across different districts of Shanghai revealed significant disparities in sample sizes (Table 3), which likely reflect variations in local ecological conditions. The highest mosquito abundance was observed in Pudong (6244), a finding attributable to its proximity to the Jiuduansha National Wetland Nature Reserve. This area provides minimal human disturbance and offers ideal breeding habitats for mosquitoes. In contrast, the high GETV seropositivity rates in Fengxian (42.5%) and Jinshan (30.6%) are consistent with the ecological settings of these regions, which are characterized by dense rice paddies, fish ponds, and pig farms—environments that facilitate both mosquito proliferation and viral amplification. Conversely, Songjiang District, which is undergoing rapid urbanization and benefits from well-maintained tourist areas, fewer livestock farms, and strict mosquito control measures, exhibited the lowest mosquito abundance and consequently reduced virus transmission pressure. These findings highlight the crucial role of ecological factors in shaping mosquito population dynamics and arboviral transmission risks.

Among the collected mosquitoes, *Culex tritaeniorhynchus* (96.39%) and *Anopheles sinensis* (2.24%) are the dominant species affecting Shanghai racecourses, aligning with their established roles as efficient alphavirus vectors in East Asia [9]. Notably, *Culex tritaeniorhynchus* not only serves as the optimal vector for GETV transmission—a competence validated through comparative studies [27]—but also plays a significant role in transmitting JEV and WNV [28]. Additionally, collected *Culex quinquefasciatus* specimens exhibited WNV amplification capacity [29, 30]. In parallel, viral detection results across all mosquito pools revealed that only JEV and GETV were identified, with WNV remaining undetected in all pools. Although AHSV is primarily transmitted by *Culicoides* midges, the potential involvement of mosquitoes in its transmission chain warrants attention [31]. However, no AHSV was detected in the mosquito samples collected in this study, suggesting that the virus has not yet circulated among mosquito populations surrounding Shanghai racecourses. The absence of WNV in both mosquitoes and equine sera, along with no AHSV detection, suggests that WNV and AHSV pose a minimal or negligible risk of becoming endemic in Shanghai, compared to the evident circulation of GETV and JEV.

Virus isolation was attempted on six RT-qPCR-positive mosquito pool specimens. Crucially, two GETV strains (SH202201 and SH202202) were successfully isolated from *Culex tritaeniorhynchus* and *Anopheles sinensis*. This achievement not only confirms the virus's adaptation to local primary vector species but also provides key biological materials for subsequent genetic evolutionary analyses. There are many species of mosquitoes carrying GIII GETV, and the virus has been isolated from midges and 17 species of mosquitoes belonging to 5 genera [32]. Phylogenetic analysis showed that SH202201 and SH202202 belong to GIII, the same genotype as GETV strains previously isolated from Hainan, Hebei, Gansu, and Shanghai, China [8, 10, 33]. Furthermore, these strains are almost completely homologous (>99.8%) to historical Shanghai strains (SH05-6 and SH05-16), indicating stability in GETV transmission cycle in the Shanghai region. In fact, GIII is the predominant GETV genotype circulating in China; however, GETV's GIV genotype, which is highly homologous to GETV strains from Russia, Malaysia, and Thailand, was also isolated in Yunnan. This may be due to the migration of migratory birds in winter and spring, which causes the intercontinental spread of GETV, WNV, and SINV [34, 35]. In addition, the migratory flight paths of wild migratory birds require several stopovers, and they can transmit GETV to local domestic aquatic birds, the environment, and even wildlife [36]. However, studies have shown that only the GIII genotype of GETV can cause epidemic outbreaks, while the GIV genotype might be under different selective pressures, possibly involving different hosts or an undetermined transmission cycle involving mosquitoes and asymptomatic hosts [4]. This could also explain why the majority of GETV strains isolated after outbreaks in multiple regions of China belong to GIII. GIII GETV triggers outbreaks and continues to spread in China, while other genotypes pose a lower threat. Therefore, developing a vaccine specifically targeting GIII GETV is crucial for controlling the virus in the country.

The E2 protein is the most important protein in GETV infection, as its interaction with the viral nucleocapsid enables the virus to bind to cell receptors [37]. Therefore, we conducted an amino acid sequence analysis and comparison of key sites in the E2 gene of SH202201 and SH202202 with different host species and strains of various genotypes. Aims to identify potential targets for vaccine development by analyzing variations in key amino acids. We found that the E2 gene of GETV is relatively conserved within the same species in the same region. For example, the SH202201 and SH202202 strains isolated from mosquitoes in this study show almost no amino acid mutations compared to the SH05-6 and SH05-16 strains previously isolated from mosquitoes in Shanghai. However, they have five key mutations compared with the M1 strain isolated from mosquitoes in Hainan. This also reflects the conserved circulation of GETV within the same region, while mutations in different regions may represent adaptive changes driven by environmental pressures. In addition, the porcine and equine strains exhibit differences at the Y86 (H→Y in JS18) and V355 (I→V in GZ201808) positions, suggesting host-specific adaptation of GETV. Studies have shown that the 253rd residue in the E2 gene is directly correlated with viral virulence. The K253R mutation significantly reduces viral virulence, possibly due to a faster viral clearance rate in the blood associated with K253R [38]. This also provides a reasonable explanation for why only the earliest isolated GI-type MM2021 strain carries the K253R mutation, while subsequent circulating or outbreak strains do not carry this mutation. Crucially, the K253R mutation in the E2 gene has been shown to significantly attenuate viral virulence in mice, identifying it as a key target for developing live-attenuated vaccines (LAVs). Consequently, the currently circulating GIII strains, which possess the virulent-associated K253 residue, could serve as ideal backbones for introducing the K253R mutation to generate safe and effective LAVs against the predominant pathogenic genotype.

Although GIII GETV remains the predominant epidemic strain in Asia, GIV GETV has re-emerged in recent years and attracted renewed attention. Even though mosquito-derived B254 (GIV) and swine-derived GETV/SW/Thailand/2017 strains have not been associated with clinical disease [39, 40], studies have revealed an amino acid substitution in the E2 protein (L269V) in the swine GIV isolate—a mutation previously considered specific to GIII strains. Given that the E2 protein is associated with host range and pathogenicity in alphaviruses [41], this mutation may potentially enhance the epidemic potential of GIV strains. Therefore, it is crucial to develop LAVs effective against multiple genotypes. This site may also represent a key determinant of virulence and could serve as a target for constructing attenuated vaccines.

Serological analysis revealed that the overall seroprevalence rates of GETV and JEV in racehorses were 28.6% (52/182) and 12.6% (23/182), respectively, with a distinct age-dependent upward trend peaking at 41.7% and 18.7% in individuals over 15 years old. This pattern aligns with cumulative exposure risks observed in Chinese equine populations, where prolonged environmental contact correlates with elevated infection rates in older horses. A previous study from South Korea reported that positive JEV antibodies in equine sera are associated not only with direct JEV infection but also potentially with vaccination [42]. As horses age, the frequency of regular JEV vaccinations increases accordingly, which may also account for the age-dependent pattern of JEV seropositivity.

Seasonal fluctuations further underscored mosquito vector involvement: significant seroprevalence increases occurred in autumn versus spring for GETV (45.1% vs. 12.1%) and JEV (18.7% vs. 6.6%), corresponding precisely with peak mosquito activity (June–September). This is the same as the infection pattern in Chinese pig herds, where GETV antibody rate in autumn exceeds the level in spring [26]. Also among pig herds, the main prevalence of JEV is from May to October [43]. This aligns with the global transmission trends of arboviruses—in warm seasons, viral transmission activity intensifies significantly. On a global scale, the transmission of mosquito-borne alphaviruses follows a similar seasonal pattern: months with rising temperatures accelerate the activity of vector insects, which facilitates viral spread [44].

Regional disparities in arbovirus seroprevalence revealed distinct ecological drivers. GETV showed significantly higher rates in Fengxian (42.5%) versus Pudong (15.6%) (*p* ≤ 0.05), potentially reflecting variations in vector density or agricultural irrigation practices, as observed in Malaysia, where irrigation-intensive farming amplified *Culex* mosquito populations and GETV spillover [4]. JEV exhibited no significant geographical variation (*p*  > 0.05), which may be consistent with uniform vaccine coverage and ubiquitous mosquito vectors.

The absence of significant gender-based differences in seroprevalence—observed for GETV and JEV—indicates a nonselective host infection pattern across arboviruses. This uniformity aligns with Japanese findings where *Culex tritaeniorhynchus* drives seasonal transmission without gender preference [12], confirming that mosquito biting behavior rather than host biological factors governs exposure risk.

Taken together, this integrated surveillance suggests that GETV and JEV may cocirculate among racehorses in Shanghai, with GETV emerging as the primary arboviral threat to the local equine population. Its distinct epidemiological profile features significant age-dependent seroprevalence, pronounced seasonal fluctuations, and geographical heterogeneity. The successful isolation of two endemic GETV strains, coupled with E2 protein conservation and host-specific adaptive signatures, confirms stable enzootic cycles in the local mosquito–equine ecosystem. Furthermore, while JEV exhibits lower equine seroprevalence (12.6%), sustained vigilance through vaccination protocols remains essential to maintain protective efficacy.

Based on the current prevalence of GETV and JEV among racehorses in Shanghai, implementing a multi-faceted prevention and control strategy is crucial. Surveillance should be intensified during high-risk seasons (autumn) and in high-risk areas (such as Fengxian and Jinshan Districts), incorporating mosquito trapping and testing alongside serological monitoring of horses. Furthermore, targeted vector control measures are essential. This includes environmental management to eliminate or control mosquito breeding sites (e.g., stagnant water bodies), application of larvicides in aquatic environments, and regular use of adulticides during peak mosquito activity periods to reduce vector populations and interrupt virus transmission. In addition to vector-focused strategies, vaccination remains a critical measure for protecting equine health against both GETV and JEV.

Overall, the threat from WNV and AHSV to Shanghai's racehorses is negligible, given their absence in this study. However, the risk of introduction remains non-zero. With the increasing number of equestrian events in Shanghai, imported horse populations from overseas may still carry related pathogens. Additionally, the wetland parks in Shanghai's Pudong district serve as critical stopover sites for migratory birds along the East Asian–Australasian Flyway, significantly elevating the risk of WNV introduction. Previous studies have indeed shown that birds in Shanghai have been infected with WNV [45]. Therefore, despite the absence of current endemicity, sustained vigilance through preventive measures is warranted. Specifically, it is essential to enhance the pathogen screening of imported horses, intensify vector biological control, and systematically conduct dynamic surveillance of relevant wetlands and migratory birds. These measures will help to reduce the risk of viral introduction and interrupt potential transmission chains.

## 5. Conclusion

This study confirms that GETV constitutes the primary arboviral threat to racehorses in Shanghai, as evidenced by its high seroprevalence (28.6%) in equine sera and the successful isolation of two viral strains (SH202201 and SH202202) from mosquito vectors. Additionally, JEV is also circulating among Shanghai's racehorses and mosquitoes; despite its relatively low seroprevalence, the potential threat it poses remains nonnegligible. Therefore, while prioritizing enhanced surveillance and control measures for GETV, it is equally crucial to implement concurrent interventions targeting JEV. Notably, WNV and AHSV—both mosquito-borne—were not detected in this study, indicating that these two viruses currently pose a minimal threat to Shanghai's equine population. However, disease prevention and control require complacency to be avoided, making the sustained surveillance of WNV and AHSV imperative.

## Figures and Tables

**Table 1:** Oligonucleotide primers and fluorogenic probes used in the RT-qPCR assay.

Virus	Location	Forward primer (5′→3′)	Reverse primer (5′→3′)	Probe (5′→3′)
GETV [23]	E2	AAGTGGCAGTACACCTCCTC	GTGGAGTTGGTCAGAGGGAA	^HEX-^AGAGCCGACCAGTTGTCTCGCA^-BHQ−1^
JEV [23]	NS1	GGGCCTTCTGGTGATGTTT	AAACCGCAGGAATVGTCAAT	^FAM-^TCGCAAGAGGTGGACGGCCA^-BHQ−1^
WNV [24]	NS5	AACCCCAGTGGAGAAGTGGA	TCAGGCTGCCACCCAAA	^CY5-^CGATGTTCCATACTCTGGCAAACG^-BHQ2^
AHSV [25]	NS1	CGCAATCTTCGGATGTAAGC	CACATACCTTGGATCTCTG	^VIC-^TCGCCATCCTCATCATCG-^-BHQ−1^

**Table 2:** Shanghai equine GETV, JEV, WNV, and AHSV serological epidemiological survey and antibody testing, 2022.

Characteristic		No. of samples	GETV		JEV		WNV		AHSV	
			No. of positive samples	Positivity rate (%)	No. of positive samples	Positivity rate (%)	No. of positive samples	Positivity rate (%)	No. of positive samples	Positivity rate (%)
Age (years)	1–5	38	12	31.6	4	10.5	0	0	0	0
	5–10	52	12	23.1	7	13.5	0	0	0	0
	10–15	36	9	25.0	5	13.9	0	0	0	0
	>15	12	5	41.7	3	25.0	0	0	0	0
	Unknown	44	14	31.8	4	9.1	0	0	0	0

Gender	Male	69	19	27.5	9	13.0	0	0	0	0
	Female	113	33	29.2	14	12.4	0	0	0	0

Season	Spring	91	11	12.1^a^	6	6.6^a^	0	0	0	0
	Autumn	91	41	45.1^b^	17	18.7^b^	0	0	0	0

Area	Songjiang	48	11	22.9^a^	8	16.7	0	0	0	0
	Fengxian	40	17	42.5^b^	3	7.5	0	0	0	0
	Pudong	32	5	15.6^a^	5	15.6	0	0	0	0
	Jinshan	62	19	30.6^a^	7	11.3	0	0	0	0

Total		182	52	28.6	23	12.6	0	0	0	0

*Note:* Identical lowercase letters in the same column indicate no significant difference (*p*  > 0.05), while different lowercase superscript letters indicate a significant difference (*p* ≤ 0.05).

**Table 3:** Distribution of mosquitoes on horse farms in Shanghai, China.

Location	Site	Mosquito species				Total percent of the sites
		*Culex tritaeniorhynchus*	*Culex quinquefasciatus*	*Anopheles sinensis*	Other mosquito species	
Songjiang No. (%)	SJ1	505 (98.63%)	0 (0.00%)	2 (0.39%)	5 (0.98%)	512 (4.60%)
	SJ2	680 (97.42%)	0 (0.00%)	1 (0.14%)	17 (2.44%)	698 (6.27%)

Fengxian No. (%)	FX1	1174 (91.72%)	14 (1.09%)	87 (6.80%)	5 (0.39%)	1280 (11.49%)

Pudong No. (%)	PD1	5450 (97.60%)	31 (0.56%)	101 (1.81%)	2 (0.04%)	5584 (50.13%)
	PD2	628 (95.15%)	17 (2.58%)	14 (2.12%)	1 (0.15%)	660 (5.92%)

Jinshan No. (%)	JS1	1201 (96.93%)	16 (1.29%)	17 (1.37%)	5 (0.40%)	1239 (11.12%)
	JS2	1100 (94.26%)	38 (3.26%)	27 (2.31%)	2 (0.17%)	1167 (10.48%)

Total No. (%)	—	10738 (96.39%)	116 (1.04%)	249 (2.24%)	37 (0.33%)	11140 (100.00%)

**Table 4:** Distribution of sample pools grouped by mosquito species and collection locations at horse farms in Shanghai, China.

Location	Site	No. of sample pools				
		*Culex tritaeniorhynchus*	*Culex quinquefasciatus*	*Anopheles sinensis*	Other mosquito species	Total
Songjiang	SJ1	5	0	1	1	7
	SJ2	6	0	1	1	8

Fengxian	FX1	12	1	1	1	15

Pudong	PD1	55	1	1	1	58
	PD2	7	1	1	1	10

Jinshan	JS1	12	1	1	1	15
	JS2	7	1	1	1	10

Total	—	104	5	7	7	123

**Table 5:** RT-qPCR detection results for four viruses in mosquito pool samples from horse farms in Shanghai, China.

Location	Site	GETV positive				JEV positive				WNV positive				AHSV positive		
		Sample pools	Mosquito species	RT-qPCR(ct)		Sample pools	Mosquito species	RT-qPCR(ct)		Sample pools	Mosquito species	RT-qPCR(ct)		Sample pools	Mosquito species	RT-qPCR(ct)
Songjiang	SJ1	—	—	—		SJ1-4	*Culex tritaeniorhynchu*s	32.18 (+)		—	—	—		—	—	—
	SJ2	—	—	—		—	—	—		—	—	—		—	—	—

Fengxian	FX1	FX1-6	*Culex tritaeniorhynchu*s	24.49 (+)		—	—	—		—	—	—		—	—	—

Pudong	PD1	PD1-32	*Culex tritaeniorhynchus*	33.91 (+)		PD1-22	*Culex tritaeniorhynchu*s	31.29 (+)		—	—	—		—	—	—
		PD1-45	*Culex tritaeniorhynchus*	34.28 (+)												
		PD1-57	Anopheles sinensis	22.35 (+)												
	PD2	—	—	—		—	—	—		—	—	—		—	—	—

Jinshan	JS1	—	—	—		JS1-9	*Culex tritaeniorhynchu*s	33.84 (+)		—	—	—		—	—	—
	JS2	—	—	—		—	—	—		—	—	—		—	—	—

**Table 6:** Amino acid differences between SH202201 and SH202202 and other classical GETV strains.

Strain name	GenBank number	Group	Host	Year	Amino acid position																						
					3	8	24	36	50	86	90	109	122	205	207	253	269	314	323	355	368	373	377	378	380	407	409
SH202201	PQ537125	Group Ⅲ	Mosquito	2022	T	V	D	K	V	H	V	D	T	S	N	K	V	V	D	I	A	A	V	V	S	V	V
SH202202	PQ537126	Group Ⅲ	Mosquito	2022	T	V	D	K	V	H	V	D	T	S	N	K	V	V	D	I	A	A	V	V	S	V	V
SH05-6	EU015066	Group Ⅲ	Mosquito	2005	T	V	D	K	V	H	V	D	T	S	N	K	V	V	D	I	A	A	V	V	S	V	V
SH05-16	EU015068	Group Ⅲ	Mosquito	2005	T	V	D	K	V	H	V	D	T	S	N	K	V	V	D	I	A	A	V	V	S	V	V
JS18	MT210319	Group Ⅲ	Pig	2018	T	V	D	K	V	**Y**	V	D	T	S	N	K	V	V	D	**V**	A	A	V	V	S	V	V
HuN1	MF741771	Group Ⅲ	Pig	2017	T	V	D	K	**I**	H	V	D	T	S	**H**	K	V	V	D	I	A	A	**A**	**I**	S	**I**	V
M1	EU015061	Group Ⅲ	Mosquito	2008	T	V	D	K	V	H	V	**G**	T	**R**	N	K	**L**	V	D	I	**V**	**C**	V	V	S	V	V
GZ201808	MK487997	Group Ⅲ	Horse	2018	T	V	D	K	V	H	**Y**	D	T	S	N	K	V	V	D	**V**	A	A	V	V	S	V	V
Rbsq202206	OP593308	Group Ⅳ	Squirrel	2022	T	V	D	K	V	**Y**	V	**N**	T	**N**	N	K	V	V	D	I	A	A	V	V	S	V	V
M 6-Mag 132	MW410934	Group Ⅱ	Mosquito	1956	T	V	D	K	V	H	V	D	T	S	N	K	**L**	V	D	I	A	A	V	V	S	V	V
MM2021	MW404214	Group Ⅰ	Mosquito	1955	**I**	**I**	**N**	**R**	V	H	**A**	D	**I**	S	N	**R**	**L**	**A**	**E**	I	A	A	V	V	**T**	V	**A**

*Note:* The bold indicates differential amino acids.

## Data Availability

The data that support the findings of this study are available from the corresponding author upon reasonable request.
